# Characterization of resistance to pine wood nematode infection in *Pinus thunbergii *using suppression subtractive hybridization

**DOI:** 10.1186/1471-2229-12-13

**Published:** 2012-01-24

**Authors:** Tomonori Hirao, Eitaro Fukatsu, Atsushi Watanabe

**Affiliations:** 1 Forest Bio-research Center, Forestry and Forest Products Research Institute, 3809-1 Ishi, Juo, Hitachi, Ibaraki 319-1301, Japan; 2 Kyushu Regional Breeding Office, Forest Tree Breeding Center, Forestry and Forest Products Research Institute, 2320-5 Suya, Goshi, Kumamoto 860-0081, Japan; 3 Forest Tree Breeding Center, Forestry and Forest Products Research Institute, 3809-1 Ishi, Juo, Hitachi, Ibaraki 319-1301, Japan

## Abstract

**Background:**

Pine wilt disease is caused by the pine wood nematode, *Bursaphelenchus xylophilus*, which threatens pine forests and forest ecosystems worldwide and causes serious economic losses. In the 40 years since the pathogen was identified, the physiological changes occurring as the disease progresses have been characterized using anatomical and biochemical methods, and resistant trees have been selected via breeding programs. However, no studies have assessed the molecular genetics, e.g. transcriptional changes, associated with infection-induced physiological changes in resistant or susceptible trees.

**Results:**

We constructed seven subtractive suppression hybridization (SSH) cDNA libraries using time-course sampling of trees inoculated with pine wood nematode at 1, 3, or 7 days post-inoculation (dpi) in susceptible trees and at 1, 3, 7, or 14 dpi in resistant trees. A total of 3,299 sequences was obtained from these cDNA libraries, including from 138 to 315 non-redundant sequences in susceptible SSH libraries and from 351 to 435 in resistant SSH libraries. Using Gene Ontology hierarchy, those non-redundant sequences were classified into 15 subcategories of the biological process Gene Ontology category and 17 subcategories of the molecular function category. The transcriptional components revealed by the Gene Ontology classification clearly differed between resistant and susceptible libraries. Some transcripts were discriminative: expression of antimicrobial peptide and putative pathogenesis-related genes (e.g., PR-1b, 2, 3, 4, 5, 6) was much higher in susceptible trees than in resistant trees at every time point, whereas expression of PR-9, PR-10, and cell wall-related genes (e.g., for hydroxyproline-rich glycoprotein precursor and extensin) was higher in resistant trees than in susceptible trees at 7 and 14 dpi.

**Conclusions:**

Following inoculation with pine wood nematode, there were marked differences between resistant and susceptible trees in transcript diversity and the timing and level of transcripts expressed in common; in particular, expression of stress response and defense genes differed. This study provided new insight into the differences in the physiological changes between resistant and susceptible trees that have been observed in anatomical and biochemical studies.

## Background

Pine wilt disease is caused by the pine wood nematode (PWN), *Bursaphelenchus xylophilus*, and was first reported by Tokushige and Kiyohara [1]; this disease threatens pine forests and forest ecosystems around the world and causes significant economic losses [2]. Pine wilt disease is a chronic problem in pine forests (*Pinus thunbergii *and *Pinus densiflora*) in Japan, where approximately 40,000,000 m^3 ^of pine forests have been blighted by the PWN [3]. Over the past 40 years, public administration and central, prefectural, and city governments have attempted to stem the spread of PWN and pine wilt disease by controlling the pine sawyer beetle (*Monochamus *sp.), the vector of PWN, with insecticides and cutting down infected trees. Additionally, national and prefectural forestry institutes have established breeding programs to develop resistant pine varieties. A breeding project to develop pine varieties resistant to pine wilt disease was started in 1978 in western Japan, and related projects were promoted throughout Japan, excluding Hokkaido Island, as the damage spread. In this breeding project, trees are screened for resistance using an artificial inoculation test that follows a strict protocol; during the first breeding program, which ran from 1978 to 1984, 92 resistant *P. densiflora *individuals were selected from 11,000 candidate trees, and only 16 resistant *P. thunbergii *individuals were selected from 14,000 candidate trees. The breeding projects continued, and 204 resistant *P. densiflora *and 90 resistant *P. thunbergii *individuals were generated. Resistant *P. densiflora *(n = 92) and resistant *P. thunbergii *(n = 16) were ranked with regard to resistance (levels 1-5) based on the survival rate of openly pollinated progeny following inoculation; higher survival rates are thought to indicate greater resistance. Average rates of survival of openly pollinated progeny from the selected pines (i.e., resistant trees) were 64% for *P. densiflora *and 53% for *P. thunbergii*, which was respectively 16% and 40% higher than for unselected populations [4].

Since the causative pathogen was identified [1], many researchers have characterized the physiological changes associated with progression of pine wilt disease, and by the mid-1990s, more than two thousand papers on the disease had been published [5]. Many symptoms associated with PWN infection, including decreased photosynthesis, denaturation of xylem and cortex parenchyma cells, traumatic resin canal formation, cambium destruction, production of phytotoxic substances and ethylene, reduced water potential and transpiration in leaves and heat pulse velocity have been studied (for review, see reference [6]). Based on the anatomical and biochemical evidence gathered during these 40 years, development of symptoms is thought to occur in two stages, early and advanced stages [6]. In the early stage, a small number of nematodes migrate and symptoms such as necrosis and destruction of cortex and phloem tissue and cambium, destruction of cortex resin canals, formation of wound periderm in cortex parenchyma around resin canals, and ethylene release all occur near the inoculation site. In the advanced stage, ethylene production is enhanced and coincides with the broad destruction of cortex parenchyma, cambial destruction, and cavitation-induced embolism of the tracheids in the xylem. The cavitation-induced embolism causes a decrease in leaf water potential and cessation of photosynthesis. After cessation of photosynthesis, severe symptoms develop rapidly with a burst in the nematode population; this population increase results in wilting and eventual tree death. Resistance against PWN infection depends on stopping the symptoms at the early stage or preventing the progression of infection to the advanced stage. While the physiological changes that occur as pine wilt disease progresses have been characterized anatomically and biochemically, molecular genetic events such as changes in transcript profiles that may be associated with the physiological changes in either resistant or susceptible trees remain poorly understood.

Recently, the gene expression profiles of resistant (resistant variety of *P. thunbergii *'Sendai-290'; resistant rank 1) and non-resistant Japanese black pine (*P. thunbergii*) trees were assessed using the LongSAGE technique on stems collected 3 days after PWN inoculation [7]. The researchers found that catalase and proteins in the dienelactone hydrolase family were highly expressed in resistant trees, but not non-resistant trees, whereas pathogenesis-related (PR)-1, 2, 3, leucoanthocyanidin dioxygenase and cell wall-related genes were expressed at higher levels in the non-resistant trees. Although the study assessed a difference of one time point in the defense responses of resistant and non-resistant *P. thunbergii *following PWN inoculation, the defense response is continuous, and the differences in resistance and susceptibility do not depend only upon qualitative differences in the activated defense genes, but also on differences in the timing and magnitude of their expression [8]. To characterize the differences in transcript profiles of resistant and susceptible trees as pine wilt symptoms develop, it is necessary to sample resistant and susceptible individuals over a defined time course.

Subtractive suppression hybridization (SSH) is a powerful tool for gene expression profiling that effectively identifies genes differentially expressed under different conditions or in different tissues [9]. This method is relatively simple and easy, it can be used with species for which there is little or no genomic information, and the cDNAs isolated are typically longer than 100 bp and can be effectively annotated using comparative genomics (e.g., BLAST analysis). This method is often used to isolate plant genes specifically expressed in response to pathogen infection and to identify differences in the transcript profiles of infected resistant and susceptible plants [10-14]. SSH selection reduces the cloning of abundantly expressed housekeeping genes or genes commonly expressed in both "tester" and "driver" samples, and therefore normalizes the expressed cDNA profiles during library construction. As a result, SSH selection significantly enhances the chances of cloning differentially expressed genes.

The goal of this study was to identify differences in the transcript profiles of PWN-inoculated *P. thunbergii *to understand the difference in the defense responses of resistant and susceptible individuals. Three important experimental design elements enhanced the clarity and relevance of our findings: 1) We used the most resistant variety of *P. thunbergii*, 'Namikata-73'; resistant rank 5. 2) We sampled inoculated trees (both resistant and susceptible) 1, 3, and 7 dpi before any macroscopic changes usually occur; resistant trees were also sampled 14 dpi, when macroscopic changes are usually evident. 3) We used SSH, a powerful approach for identifying differentially expressed genes regardless of their abundance.

## Results

### Analysis of sequences in SSH libraries

Six subtractive libraries constructed from samples taken at three time points, 1, 3, and 7 days dpi, were used to identify genes that are differentially expressed in resistant and susceptible trees as disease symptoms develop following PWN inoculation. In addition, a seventh library contained genes expressed predominantly in resistant trees at 14 dpi compared to susceptible trees at 7 dpi (see Methods). We sequenced nucleotides from the 5'-end of 3,299 cDNA inserts that were recovered in the seven libraries (Table 1). Insert length varied from 100 to 780 bp; the median length ranged from 366 to 429 bp, depending on the library. The redundancy within each library varied from 3.9% to 27.2% in libraries from resistant trees and from 28.4% to 72.8% from susceptible trees. The obtained non-redundant expressed sequence tags (ESTs) ranged from 351 to 435 in libraries from resistant trees and from 138 to 315 in libraries from susceptible trees. Importantly, the overlap between the two libraries at each time point was extremely low; for example, 0.4% of the cDNA inserts were shared between libraries from resistant and susceptible trees sampled at 1 dpi. Similarly, only 3.7% of the cDNA inserts were shared by the library from resistant trees sampled at 14 dpi and from susceptible trees sampled at 7 dpi. This high level of specificity is expected from efficient SSH procedures.

**Table 1:** Characterization of seven SSH libraries

**SSH libraries**^**a**^		Total ESTs sequences	Length of sequence (bp)	Contigs	Singletons	Non-redundant ESTs	Redundant (%)	Unique (%)	**Hit rate using the BLAST program in Blast2GO tool (%)**^**b**^					Hit rate using the local BLAST program (%)	Automatic annotation rate (%)	**Manual annotation rate (%)**^**d**^	Total annotation rate (%)
											
Tester	Drivers								**blastx to nrDB**^**1)**^	**tblastx to nrDB**^**2)**^	**tblastx to estDB**^**3)**^	**blastn to estDB**^**4)**^	no hit	**blastn to PGI**^**C**^			
R_1dpi	S_1dpi	437	102-775	14	406	420	3.9	96.1	71.0	17.1	7.0	3.8	1.1	97.1	65.8	9.7	75.5

S_1dpi	R_1dpi	449	108-777	52	214	266	40.8	59.2	79.7	10.8	7.5	1.6	0.4	96.9	73.3	6.9	80.2

R_3dpi	S_3dpi	455	102-775	43	344	387	14.9	85.1	72.4	15.7	7.5	3.5	0.9	97.6	65.1	10.6	75.7

S_3dpi	R_3dpi	440	101-760	43	272	315	28.4	71.6	81.1	12.2	4.1	1.4	1.2	96.4	77.0	7.4	84.4

R_7dpi	S_7dpi	528	100-767	41	394	435	17.6	82.4	79.5	10.3	6.9	3.0	0.3	98.0	74.1	7.8	81.9

S_7dpi	R_7dpi	508	104-780	48	90	138	72.8	27.2	91.8	5.9	1.7	0.6	0.0	99.4	84.7	6.3	91.0

R_14dpi	S_7dpi	482	106-764	52	299	351	27.2	72.8	73.8	15.3	7.2	3.3	0.4	98.0	66.7	11.0	77.7

^a ^R = resistant, S = susceptible, dpi = days post-inoculation

^b ^The edited sequences in each library were compared to the NCBI database using the following algorithms: 1) blastx program against the non-redundant protein database of NCBI with a threshold value of e-6, 2) tblastx program against the nucleotide database of NCBI with a threshold value of e-6, 3) tblastx program against the EST database of NCBI with a threshold value of e-6, 4) blastn program against the EST database of NCBI with a threshold value of e-10

^c ^The edited sequences in each library were compared to the PGI database (DFCI Pine Gene Index, release 8.0) using the blastn program with a threshold value of e-10

^d ^Manual annotation based on the annotated gene information in the PGI database

### Functional classification of the expressed genes in each SSH library and identification of differentially accumulated genes

In order to annotate the putative gene and functional Gene Ontology (GO) categories for the transcripts of each SSH library, we compared non-redundant ESTs of each SSH library with the GenBank non-redundant and EST databases using the search programs blastx, tblastx, and blastn using the Blast2GO program [15] (Additional file 1). The transitional hit rate of the BLAST analysis and the annotation rate per library are indicated in Table 1. For each library, 98% or more of the clones were matched in a BLAST search using the nr and EST databases at the National Center for Biotechnology Information (NCBI). Similarly, 97% or more of the clones in each library were matched in a BLAST search (blastn) using the Dana-Farber Cancer Institute *Pinus taeda *Gene Index (DFCI PGI, release 8.0). The annotation rates for GO terms resulting from automated and manual annotation varied from 75.45% to 91.00% per library.

**Figure 1:**
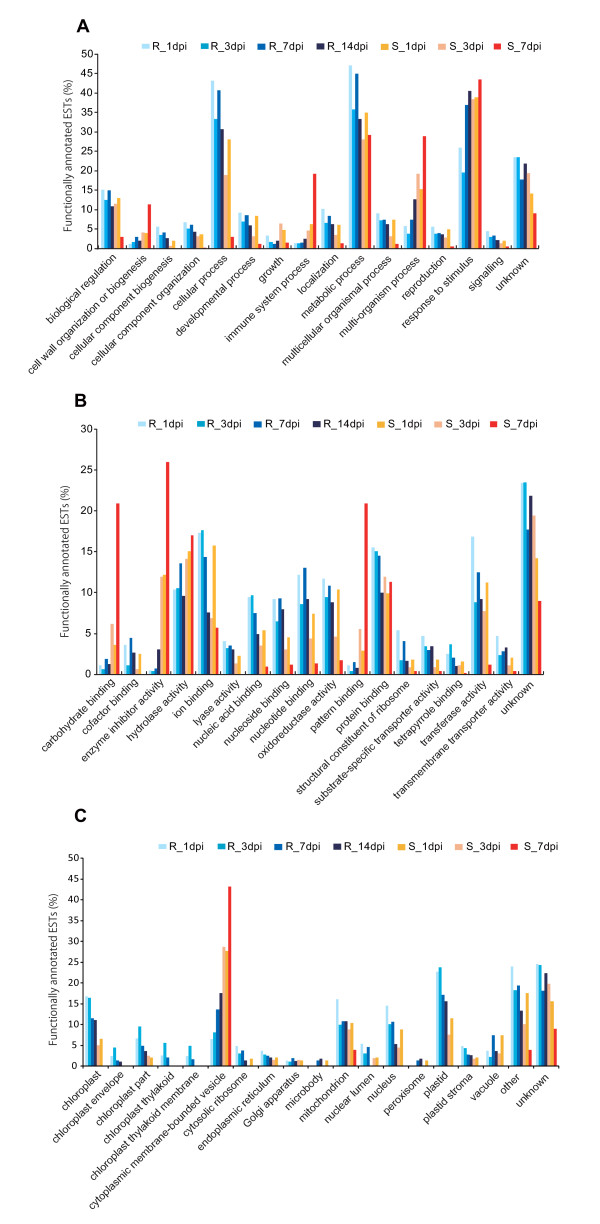
**Gene Ontology mapping for SSH libraries from resistant and susceptible trees**. EST distribution in the categories (**a**) Biological process, (**b**) Molecular function, and (**c**) Cellular component.

Non-redundant ESTs recovered in the seven libraries were classified by function into the three principal Level 1 GO categories: biological process, molecular function, and cellular component. Furthermore, the biological process category was split into 24 subcategories at Level 2, molecular function was split into 61 subcategories at Level 3, and cellular component was split into 39 subcategories at Level 7. The level presented herein corresponds to the depth of hierarchical categories within each principal GO category, and higher levels represent more general classifications. The classification for biological process, divided into 15 major subcategories and 24 lower-level subcategories, is shown in Figure 1a. Of the 15 subcategories under biological process, the subcategory of response to stimulus was present at a higher percentage in the susceptible libraries at each time point, and three categories-cell wall organization or biogenesis, immune system process, multi-organism process-were present at a higher percentage in the susceptible libraries than in the resistant libraries at each time point. In contrast, transcripts related to cellular process and metabolic process were present at a higher percentage in the resistant libraries. In addition, the category of response to stimulus was present at a higher percentage even in the resistant libraries at 7 and 14 dpi. The classification for molecular function, divided into 17 major subcategories and 61 lower-level subcategories, is shown in Figure 1b. At each time point, susceptible trees expressed more transcripts in the four subcategories of carbohydrate binding, enzyme inhibitor activity, hydrolase activity, and pattern binding than did resistant trees, whereas transcripts related to several molecular binding categories such as ion binding and protein binding and to several molecular activity categories such as oxidoreductase activity and transferase activity were present at a higher percentage in the resistant libraries. The classification for cellular component, divided into 18 major subcategories and 39 lower-level subcategories, is shown in Figure 1c. At each time point, susceptible trees expressed more transcripts in the cytoplasmic membrane-bounded vesicle category than did resistant trees. In contrast, the transcripts related to organelle categories such as chloroplast, plastid and mitochondrion were present at a higher percentage in the resistant libraries at each time point.

In order to evaluate the specificity and diversity of the transcripts that were specific in a time course from infected resistant and susceptible trees, ESTs were selected from each SSH library (Table 2, Additional file 2). The libraries from susceptible infected trees showed very limited transcript diversity and were chiefly composed of PR proteins, such as basic PR-1, PR-2 (beta-1,3-glucanase), PR-3 (class I, IV chitinase), PR-4 (chitinase type I & II), PR-5 (thaumatin-like protein), PR-6 (type II proteinase inhibitor family protein), and antimicrobial peptide. In contrast, the ESTs from resistant trees were more diverse. Transcripts encoding metallothionein-like protein, heat shock protein 70 (HSP70), xyloglucan endotransglycosylase (XET), and cytochrome P450 (CYP450) were discernible in the resistant library at 1 dpi and 3 dpi. The libraries from resistant trees sampled at 7 dpi and 14 dpi had transcripts encoding PR-5, PR-9 (peroxidase), PR-10 (ribonuclease-like), hydroxyproline-rich glycoprotein (HRGP) precursor, and extensin.

**Table 2:** Characteristic ESTs within each SSH library

Putative gene categories based on BLAST annotations	Number of ESTs from each SSH library*						
	
	R_1dpi	R_3dpi	R_7dpi	R_14dpi	S_1dpi	S_3dpi	S_7dpi
PR-1 family	0	0	1	0	2	6	1

PR-2 family (beta-1, 3-glucanase)	1	0	1	2	7	12	21

PR-3 family (class i chitinase)	2	1	4	1	6	2	1

PR-3 family (class iv chitinase)	1	0	2	5	9	9	58

PR-4 family	1	0	1	1	14	5	56

PR-5 family (thaumatin-like)	0	0	12	62	55	52	129

PR-6 family (proteinase-inhibitor)	0	0	2	10	56	52	130

PR-9 family (peroxidase)	1	0	5	1	3	5	0

PR-10 family (ribonuclease-like)	0	8	57	1	24	11	20

Antimicrobial peptide	0	0	0	1	27	13	8

Cytochrome P450	4	4	1	0	0	0	0

Extensin	0	0	0	27	10	5	4

Heat Shock Proteins	10	4	3	22	1	0	0

Hydroxyprolinerich glycoprotein precursor	0	0	6	16	1	1	1

metallothionein-like protein	7	26	4	0	1	0	0

Xyloglucan endotransglycosylase	5	4	2	0	0	1	0

Rate of occupying a library (%)	6.8	10.1	18.8	30.5	47.7	39.2	84.0

* R = resistant, S = susceptible, dpi = days post-inoculation

### Validation of differential expression using selected SSH clones and quantitative real-time PCR (qRT-PCR)

**Figure 2:**
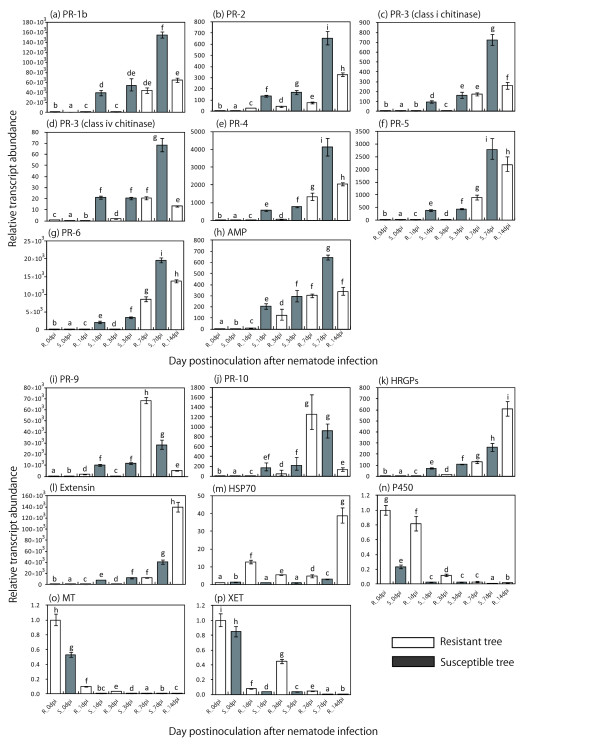
**Quantitative real-time PCR of transcripts differentially expressed in resistant and susceptible trees following PWN inoculation**. The putative functional genes from (a) to (h) were clearly discernible ESTs in susceptible SSH libraries. The putative functional genes from (i) to (p) were clearly discernible ESTs in resistant SSH libraries. Elongation factor 1-alpha (EF1a) was used as the reference gene, and the data were calibrated relative to the transcript levels in resistant trees prior to nematode inoculation (at 0 days). The data are presented as the mean ± S.D. of three replicates. Means designed by the same letter did not significantly differ at *P *< 0.05 according to Tukey's HSD test.

To validate the results of the SSH procedures, expression of 16 ESTs recovered in one or more of the seven libraries was assayed using qRT-PCR; samples for the qRT-PCR analysis were collected from resistant and susceptible trees at 0, 1, 3, 7, and 14 dpi. (Figure 2). Furthermore, expression of 16 ESTs of mock samples at each time points was also assayed using qRT-PCR to monitor expression changes induced either by PWN infection or by cutting (Additional file 3). The results of the qRT-PCR analysis and SSH were consistent.

Expression of 12 of 16 ESTs was clearly upregulated following inoculation with PWN. Among the upregulated ESTs, expression of putative PR-1b, PR-2, PR-3 (class I & IV chitinase), PR-4, PR-5, PR-6, and antimicrobial peptide was much higher in the susceptible trees than in the resistant trees at each time point. Expression of these ESTs was much higher in susceptible trees at 1 dpi and expression had increased further by 7 dpi. In the resistant trees, expression of these ESTs was at a relatively low level at 1 dpi and 3 dpi, but was abundant at 7 dpi and 14 dpi. ESTs encoding PR-9, PR-10, HRSP, and extensin were discernible in resistant SSH libraries. Expression of PR-9 and PR-10 was much higher in the susceptible trees at 1 dpi and 3 dpi; however, levels of these two transcripts were much higher in resistant trees at 7 dpi, though it was lower at 14 dpi than 7 dpi. HRGP and extensin transcript accumulation in resistant trees was high at 14 dpi, although expression of both ESTs was higher in susceptible trees than in resistant trees at 1 dpi, 3 dpi, and 7 dpi.

Based on qRT-PCR analysis, three ESTs--CYP450, metallothionein-like protein, and XET--were downregulated in both resistant and susceptible trees after PWN inoculation, with greater downregulation in susceptible trees. Transcript levels of these three ESTs decreased in susceptible trees within 1 dpi, and were even lower at 3 and 7 dpi. The levels of these ESTs in resistant trees at 7 and 14 dpi were similar to those in susceptible trees at 7 dpi. The transcript level of HSP70 was lower than the other downregulated genes, and its decrease was more moderate in resistant trees, with an expression pattern similar to that of mock samples.

## Discussion

We sequenced cDNAs in seven SSH libraries to characterize transcriptional differences between resistant and susceptible *P. thunbergii *trees in response to inoculation with PWN. In susceptible trees, transcript diversity was statistically lower than in resistant trees at the three time points jointly tested, 1, 3, and 7 dpi. In susceptible trees, several transcripts encoding pathogenesis related proteins were present at a higher constitutive level than in resistant trees. In resistant trees at 14 dpi, several transcripts encoding cell wall proteins were identified. The results of the SSH approach were validated by qRT-PCR. We clearly demonstrated that transcript composition, temporal changes, and levels of gene expression involved in the stress/defense response to PWN inoculation in resistant trees differs from susceptible trees.

There was a significant difference in transcript diversity between resistant and susceptible trees after infection with PWN. The percentage of unique sequences in susceptible libraries ranged from 71.59 to 27.17%, whereas the percentage of unique sequences in resistant libraries ranged from 72.82 to 96.11%. Although transcript diversity of the susceptible library at 3 dpi was higher than the susceptible libraries at 1 and 7 dpi, transcript diversity in resistant libraries was higher than susceptible libraries at all time points after PWN infection. According to the GO classification of the differentially expressed transcripts, a large percentage in susceptible trees was involved in stress/defense response categories such as the response to stimulus, multi-organism process, and immune system process within the biological process category and the enzyme inhibitor activity, carbohydrate binding, pattern binding, and hydrolase activity within the molecular function category. Furthermore, a large percentage of transcripts in the cellular component category were in the cytoplasmic membrane-bounded vesicle subcategory. On the other hand, a large percentage of transcripts in resistant SSH libraries was categorized into the cellular process, metabolic process and response to stimulus subcategories of the biological process category and in the ligand binding and transferase activity subcategories of the molecular function category. Many transcripts recovered in libraries from resistant trees were assigned to the cellular component subcategory and further classified into organelle subcategories of plastid, chloroplast, and mitochondrion. The difference in transcript diversity between libraries from resistant and susceptible trees revealed by the GO classification indicated a qualitative difference in the stress/defense response of resistant and susceptible trees to PWN infection.

Resistance and susceptibility do not depend only upon qualitative differences in the activated defense genes, but also on differences in the timing and magnitude of their expression [8]. The gene regulation patterns of pathogenesis related defense proteins such as PR-1b, PR-2, PR-3, PR-4, PR-5, PR-6 and antimicrobial peptide indicated temporal and quantitative differences between resistant and susceptible trees in response to PWN infection. In regulating the plant defense response, most pathogenesis related proteins are induced through the action of the signaling compounds salicylic acid (SA), jasmonic acid (JA), or ethylene [16]. PR-1b, PR-2 and PR-5 genes are SA-responsive genes and also indicators of systemic acquired resistance; additionally, the PR-6 gene is a JA and ethylene responsive gene [17]. Although the relationship between phytohormones and the defense response in the PWN-nematode interaction is not clear from this study, it is interesting that expression of pathogenesis related genes associated with phytohormones such as SA and JA/ethylene and with antimicrobial activities were induced more quickly and to a higher level in susceptible than in resistant trees.

The same three phytohormones, SA, JA, and ethylene, are important for both basal and R-gene mediated defense responses to foliar pathogens and insects. The JA and ethylene signaling pathways seem to work synergistically, whereas the SA and JA/ethylene signaling pathways inhibit each other, and negative cross-talk exists between SA and JA/ethylene signaling pathways [18]. In interactions between *Hero A*-resistant tomatoes and cyst nematode, expression of SA-responsive genes PR-1 and PR-5 is a hallmark of the resistant cultivar, and expression of JA-dependent PR-6 is higher in the susceptible cultivar, indicating that SA plays some role in the resistance to the nematode and that JA and ethylene in susceptible tomatoes are likely to interfere with the SA-inducible resistance pathway [19,20]. The involvement of SA in resistance and expression of SA-responsive genes in resistant plants has been observed in interactions between other plant species and nematodes (e.g., an *Arabidopsis thaliana *mutant [21] and a root knot nematode resistant peanut [13]). In our study, the higher induction of both SA-responsive genes such as PR-1b, PR-2, PR-5 and JA/ethylene-responsive genes such as PR-6 in susceptible trees indicates that the defense response mediated by these phytohormones was not very effective in controlling PWN infestations.

We detected notable levels of putative HRGPs, extensin and peroxidase (PR-9) in resistant trees at 7 dpi and 14 dpi, though expression in susceptible trees was higher than in resistant trees at 1 and 3 dpi. Cell wall-mediated resistance is the first line of plant defense against pathogens, and the components of plant cell wall are modified by production of reactive oxygen species (ROS) such as H_2_O_2 _during attack by pathogens [22]. The structural cell wall proteins extensin and HRGPs play an essential role in biotic and abiotic stress responses due to their oxidative cross-linking, which contributes to the strength of cell walls and is catalyzed by an oxidizing system based on peroxidase and H_2_O_2 _[23-25]. The cross-linking of HRGPs and extensin is involved in cell wall-mediated resistance and has a major role in arresting pathogens at the site of entry [23]; these proteins accumulate in the walls of a number of plant species during interactions with microbes [26]. A number of reports describe the response of plant cell wall HRGPs and extensin to pathogens such as viruses, bacteria, and fungi (for review, see reference [27]). In plant-nematode interactions, high extensin gene expression was observed in the cortical region of tobacco at 7 and 14 days after inoculation with root knot nematodes [28]. In *rhg1 *resistant soybean, extensin was identified as one of the genes characteristically expressed in syncytia after inoculation with soybean cyst nematode, indicating that altered cell wall composition is important in the defense response [29]. Also, in *Mi *resistant tomato, extensin and glycosyltransferase may play a role in cell wall synthesis, which is an essential defense against root knot nematode [30,31]. In anatomical studies of PWN infection, Ishida et al. [32] inoculated virulent nematode (*B. xylophilus*) isolate S6-1 and avirulent nematode (*B. mucronatus*) isolate B. m to Japanese black pine, and observed accumulation of lignin- and suberin-like substances around the resin canals in the cortex with both nematode isolates at 7 dpi. Kusumoto et al. [33] also inoculated a virulent nematode (*B. xylophilus*) isolate, Ka-4, to Japanese black pine and found more frequent accumulation of phenolic compounds around the cortex resin canals in resistant trees at 7 dpi and 14 dpi after inoculation with PWN (*B. xylophilus*), and suggested that this accumulation was a very effective defense against infection due to restricting PWN migration. Although the relationship between HRGPs or extensin and other substances in the cell wall was not examined in this study, it is possible that upregulation of expression of cell wall-related genes such as those for HRGPs or extensin and oxidative cross-linking of these proteins by peroxidases plays a role in the effective defense response of trees resistant to PWN infection at 7 dpi and 14 dpi.

PR-10 was also one of the characteristically significantly upregulated genes in libraries in the 7 dpi subtraction library from resistant trees. Although the biological function of PR-10 remains unclear, many PR-10 genes are upregulated when plants are exposed to abiotic stresses, such as SA, CuCl_2_, H_2_O_2_, cold, darkness and wounding [34], and biotic stresses, such as viruses [35], bacteria [34,36], fungi [37-40] and insects [41,42]. We observed synchronized expression of PR10 with peroxidase in resistant trees, which indicates this gene may be induced by ROS such as H_2_O_2_. However, PR10 (CpPRI) acts against a digestive proteinase from the root knot nematode *Meloidogyne incognita *[43]. Therefore, PR10 may act as a proteinase against cellulases, beta-1,3-glucanase, and pectate lyases secreted from PWN [44-46].

Heat shock protein (HSP) ESTs were characteristically recovered in libraries from resistant trees. In particular, stable HSP70 expression in infected resistant trees was validated by qRT-PCR. HSP family members, which consist of HSP70, HSP60 and HSP90, are required for folding of nascent proteins and intracellular transportation in addition to stress responses, and are collectively called molecular chaperones [47]. In the interaction between soybean and soybean cyst nematode, Klink et al. [48] observed the induction of HSP70 and ROS responsive genes such as lipoxygenase and superoxidase dismutase isolated from 3 dpi syncytial cells showing an incompatible response to soybean cyst nematode infection, and suggested that HSP70 may be involved in maintaining a properly functioning environment for other defense responses. In the present study, it is unclear how HSP70 is involved in the defense response to PWN infection.

Three ESTs--putatively encoding CYP450, metallothionein-like protein, and XET--were detected in resistant SSH libraries at 1 dpi and 3 dpi, which depend on the genes significantly downregulated in susceptible trees at 1 and 3 dpi. In plants, CYP450 monooxygenases play paramount roles in the synthesis of lignin intermediates, sterols, terpenes, flavonoids, isoflavonoids, furanocoumarins, and a variety of other secondary plant products [49]. In conifers, diterpene resin acids are prominent defense compounds against insect pests and pathogens in conifers [50-52], and CYP450s are involved in the formation of a suite of diterpene resin acids of conifer oleoresin; they oxidize abietadienol and abietadienal to abietic acid in vitro and in vivo [53,54]. Keeling and Bohlmann [50,51] indicated that oleoresin may contain specific components that are toxic or deterrent to insect herbivores or may affect adults or broods physiologically and thus prevent successful colonization or reproduction. The downregulation of CYP450 observed in the present study may cause a reduction in diterpene resin acids in pine trees infested with PWN. Consequently, PWN may expand its invasion and habitat, and the rapid reduction in CYP450 expression in susceptible trees may trigger PWN expansion.

Metallothioneins are involved in ROS scavenging, and in rice, downregulation of metallothionein expression is observed during the oxidative burst phase in elicitor-treated cells, and metallothionein expression is important for defense signaling [55,56]. The metallothionein expression we observed indicated that ROS accumulation and defense signaling may have been induced by 1 dpi in susceptible trees infected with PWN, whereas it may not have been induced much in resistant trees 3 dpi later; this provides evidence for rapid induction of defense response genes such as those encoding pathogenesis related proteins in susceptible trees.

XET action is thought to regulate wall loosening during turgor-driven expansion by rearranging load-bearing xyloglucan cross-links between cellulose microfibrils, and its activity and expression have been detected in growing tissues [57-64]. We found that XET was downregulated in both resistant and susceptible trees following PWN infection, and its regulation was induced relatively early, by 1 dpi. These findings suggest that the expansion of cell walls in xylem, phloem or both is inhibited by the downregulated XET during PWN infection; alternatively, the cell wall may be immobilized by the cross-linking of HRGPs or extensin.

In pine-nematode interactions, Myers [65] and Futai [66] suggested that invasion and rapid migration of a few mobile parasites through tree tissues invokes an innate hypersensitive reaction such as death of the parenchyma, production of toxins, and leakage of oleoresins and other material into tracheids. Furthermore, the population of PWN spreads throughout the whole body, and a series of hypersensitive reactions eventually leads to tree death in susceptible pine species. In this study, defense response genes, antimicrobial peptide, SA-responsive genes such as PR-1b, PR-2, PR-5 and JA/ET-responsive genes such as PR-6 were induced more quickly and to a higher level in susceptible than in resistant trees. These defense responses in susceptible trees would not be effective in controlling PWN nematode infestations, and defense signaling induced within the tree may then induce a series of hypersensitive reactions through cellular interactions that subsequently lead to death, as Myers [65] and Futai [66] suggested. In contrast, a moderate hypersensitive reaction along with upregulation of pathogenesis related genes followed by upregulation of cell wall-related genes contributing to the strength of cell walls would be a very effective defense against PWN infection, because these events might restrict PWN migration.

## Conclusions

This is the first study to assess the difference in EST transcript diversity of activated defense genes and differences in the timing and magnitude of expression of these genes between resistant and susceptible *P. thunbergii *trees following PWN inoculation. In susceptible trees after PWN inoculation, pathogenesis related genes and antimicrobial-related genes were rapidly induced to high levels within 1 dpi; this finding indicated that a hypersensitive reaction-like response was induced in susceptible trees and supported the hypothesis of Myers [63] and Futai [64]. In contrast, a moderate defense response mediated by pathogenesis related protein expression followed by significant upregulation of cell wall-related genes induced by ROS was a very effective defense against PWN infection. The ESTs generated in our study will certainly advance understanding of defense mechanisms against PWN at the transcriptional level in other varieties or other *Pinus *species.

## Methods

### Plant materials and nematode inoculation

A resistant tree of 'Namikata 73,' which is the most highly resistant variety of the 16 resistant varieties selected out of 15,000 individuals from 1990 to 1998 [4], was planted in the Forest Products Research Institute, Forest Tree Breeding Center (FFPRI-FTBC) in Ibaraki, Japan. A susceptible tree of the variety 'Kataura 1,' selected as a plus-tree for growth traits, was also in the FFPRI-FTBC. Both clones were grafts obtained from the original trees at the FFPRI-FTBC in 2005. The PWN used in this study was the Ka-4 isolate, which has been used in pine wilt disease resistance breeding projects since 2003.

Inoculation with PWN was conducted on July 1, 2007. In four susceptible clones and four resistant clones, 2 cm at the tip of the main stem was cut off, the cut edge was quickly crushed with pliers, and 10,000 nematodes that had been suspended in 100 μl sterile water were injected into the cut edge. As a mock sample, sterile water (without nematodes) was injected into the cut edge of the main stem of another four susceptible clones and four resistant clones. Stem tissue of inoculated samples and mock samples was collected 5 cm below the inoculated stem apex at 1, 3, 7, and 14 dpi. A 2 cm segment of stem was cut, frozen immediately in liquid nitrogen, and stored at -80°C.

### RNA isolation

Total RNA was isolated from 1.5 g of stem that included bark and wood tissue using the RNeasy plant mini kit (QIAGEN) following the protocol supplied by the manufacturer. RNA concentration was determined using a GeneQuant pro spectrophotometer (Amersham Biosciences). Integrity of the RNA was tested using the Agilent 2100 bioanalyzer (Agilent Technologies).

### SSH library construction, DNA sequencing, data analysis and dbEST submission

Six subtractive libraries were constructed from samples taken at three time points; specifically, two libraries--one from resistant trees and one from susceptible trees--were constructed from samples taken 1, 3, or 7 days dpi. Forward libraries containing genes expressed predominantly in resistant trees were constructed by subtracting driver RNA sampled from susceptible trees from tester RNA sampled from resistant trees, and reverse libraries containing genes expressed predominantly in susceptible trees were constructed by subtracting driver RNA sampled from resistant trees from tester RNA sampled from susceptible trees. Additionally, a seventh library was constructed by subtracting driver RNA sampled from susceptible trees 7 dpi from tester RNA sampled from resistant trees 14 dpi; the driver RNA samples were taken at 7 dpi because the susceptible trees had died by day 14. SSH libraries were constructed using a SuperSMART cDNA Synthesis kit (Clontech) and a Clontech PCR-Select cDNA Subtraction kit (Clontech). The SSH products were purified using a QIAquick PCR purification kit (QIAGEN) and ligated into the pT7Blue vector (Merck4Biosciences). Blue/white selection was conducted on plates containing ampicillin, isopropyl-D-thiogalactopyranoside and X-gal. Clones were randomly selected and single-pass sequenced using a primer that recognizes vector sequences from the 5' -end of the inserts. On -average, 500 clones were sequenced per library using an ABI 3130xl DNA Analyzer (Applied Biosystems). The resulting sequences were trimmed and edited manually to identify the cloning vector sequences, adaptor sequences used in the SSH procedure, and regions of low-quality sequence using Sequencher 4.10.1 software (Gene Codes Corp.). Quality sequences greater than 100 bp were selected for further analysis. A total of 3,299 sequences were at least 100 bp and these sequences were submitted to the GenBank EST database with the GenBank accession numbers FY841122 to FY844420. To determine the number of contigs and EST singletons in each library, the ESTs were assembled using Sequencher 4.10.1 by the requirement for at least 98% identity over each 20 bp continuous sequence.

### EST similarity search and functional assignments

A similarity search and functional annotation were performed for the EST singletons in each library using online version of the BLAST2GO program (BLAST2GO 2006; [15]). The thresholds used with the BLAST algorithms were as follows: (1) blastx comparison with the non-redundant protein database of NCBI with a threshold value of e-6; (2) tblastx comparison with the nucleotide database of NCBI with a threshold value of e-6; (3) tblastx comparison with the EST database of NCBI with a threshold value of e-6; (4) blastn comparison with the EST database of NCBI with a threshold value of e-10. Additionally, the ESTs in each library were compared with 69,968 EST sequences in the DFCI Pinus Gene index release 8.0 using an E value cut-off of e-10. The ESTs were assigned to functional categories using the Blast2GO program, and manual annotations were based on the result of BLAST analysis using the PGI database. The Blast2GO program extracts the GO terms associated with homologies identified with NCBI's QBLAST and returns a list of GO annotations represented as hierarchical categories of increasing specificity. Therefore, the "level" presented in this study corresponds to the depth of hierarchical categories in each principal GO category, with the topmost Level 1 representing the most general classification (principal GO categories) of biological process, molecular function, and cellular component.

### Real-time qRT-PCR

Primer pairs were designed for each sequence using Primer Express software v3.0 (Applied Biosystems) and following the manufacturer's guidelines for primer design (Table 3). For SYBR Green real-time RT-PCR assays, the amplification efficiency of all primer pairs was optimized with genomic DNA from resistant and susceptible trees using the StepOnePlus Real-Time PCR System (Applied Biosystems).

**Table 3:** Primers used in this study

Putative gene function	Forward primer	Reverse primer	Fragment length (bp)	**GenBank Acc**.
PR-1b family	5' -TGCCCCTTCAGGTAAATCGT-3'	5' -GCGGGTCGTAGTTGCAGATAA-3'	125	FY841927

PR-2 family (Beta-1,3-glucanase)	5' -CGACAACATTCGCCCCTTCT-3'	5' -CTGCAGCGCGGTTTGAATAT-3'	130	FY843702

PR-3 family (class I chitinase)	5' -ACCTACAGCGCCTTCATTGC-3'	5' -TGTGGTTTCATGCGACGTTT-3'	120	FY841849

PR-3 family (class Iv chitinase)	5' -CCATCGAAGCCCAGGTAATTT-3'	5' -AGCCGGGAAGCAATATTATGGT-3'	90	FY843470

PR-4 family	5' -CCCCGTTACTGTCAATTGCAT-3'	5' -AAAGCGTGACGGTGCGTATT-3'	90	FY841704

PR-5 family (thaumatin-like)	5' -GAACCAGTGCCCATACACAGTCT-3'	5' -CCTGCGGCAACGTTAAAAGTC-3'	96	FY842709

PR-6 family (proteinase inhibitor)	5' -TGCTGGCGGCATCTATTTTA-3'	5' -TAACACCTGCGCAAATGCA-3'	90	FY843534

PR-9 family (peroxidase)	5' -ACACCACCGTGCTGGACATT-3'	5' -GTGCGGGAGTCGGTGTAGAG-3'	118	FY842918

PR-10 family (ribonuclease-like)	5' -TGTCTCAAGTGGAGGCAAGGA-3'	5' -AAGCGACAATTTCAGGCAAAAC-3'	90	FY842956

Antimicrobial peptide	5' -GCGTTGCTCATACCCGTTTT-3'	5' -GCAGCACTTAGCACTGGATGAA-3'	90	FY841562

Cytochrome P450	5' -AACATGTCCTGCAGCACGAA-3'	5' -GTGCACCGCAAGTAAACCAA-3'	95	FY841345

Extensin	5' -CGAATGTAATTCCGAAGTTGCA-3'	5' -CCATCCCAAACCACCAGTCT-3'	110	FY844277

Heat shock protein 70	5' -AACACCACCATTCCCACCAA-3'	5' -CGAATTTGCCGAGCAGGTTA-3'	130	FY841300

Hydroxyproline-rich glycoprotein precursor	5' -GAGAAACTGGCACCGTCTTAGGA-3'	5' -ACCTCCCCCTCCATCTCACA-3'	140	FY843962

Metallothionein-like protein	5' -TCAGGCTGCTGCGTTATTTG-3'	5' -TGTCAGCGCAGTCACAATTTG-3'	120	FY842178

xyloglucan endotransglycosylase	5' -TCTGCGCCCCTACTTTTCC-3'	5' -AGCTGGGCGATTGATCATGT-3'	121	FY842425

Elongation factor-1 alpha	5' -GGGAAGCCACCCAAAGTTTT-3'	5' -TACATGGGAAGACGCCGAAT-3'	160	FY842441

The putative functional genes from (a) to (h) were clearly discernible ESTs in susceptible libraries. The putative functional genes from (i) to (p) were clearly discernible ESTs in resistant trees. Elongation factor 1-alpha (EF1a) was used as the reference gene, and the data were calibrated relative to the transcript levels in resistant trees prior to nematode infection (at 0 dpi). The data are presented as the mean ± S.D. of three replicates. Means designed by the same letter did not significantly differ at *P *< 0.05 according to Tukey's HSD test

RT-PCR was performed using the total RNA used to make the SSH libraries. For the mock samples at each time point and for the reference sample (without nematodes or water), total RNA was extracted from stem tissues and used for RT-PCR. Total RNA (500 ng in a final volume of 20 μL) was reverse-transcribed using the PrimeScript II 1st strand cDNA synthesis kit (TaKaRa) according to the manufacturer's protocol. Real-time quantitative PCR was performed using the *Power *SYBR Green PCR Master Mix (Applied Biosystems) on the StepOnePlus Real-Time PCR System (Applied Biosystems). PCR mixtures were prepared according to the manufacturer's instructions and contained 300 nM of both the forward and reverse gene-specific primers and 4 μL of the 20-fold diluted reverse transcription reaction (total 5 ng) in a final volume of 20 μL. All reactions were heated to 95°C for 10 min; this denaturation step was followed by 40 cycles of 95°C for 15 s and 60°C for 1 min. The PCR products were subjected to melting curve analysis; the conditions were incubation at 60-95°C with a temperature increment of 0.3°C s^-1^. Elongation factor 1-alpha was used as the reference gene for normalizing the transcript profiles. The real-time PCR data were calibrated against the transcript levels in resistant tree stem samples prior to nematode exposure; the 2^-ΔΔCt ^method was used to quantify relative transcript abundance [67]. All assays were carried out in triplicate, and the data are presented as means ± S.D. of three replicates. The specificity of each amplification was checked by melting analysis and agarose gel electrophoresis of the amplified products.

## Authors' contributions

TH prepared the RNA preparation, sequenced the ESTs, analyzed the EST data, and drafted the manuscript. EF helped with data analysis of the ESTs and prepared the DDBJ GenBank submissions. AW conceived of the project, carried out the inoculation of PWN and sampling, and helped to draft the manuscript. All authors assisted with manuscript preparation and read and approved the final draft.

## Supplementary Material

Additional file 1 **Summary of BLAST search results for ESTs among 7 SSH libraries**. Click here for file

Additional file 2 **Summary of BLAST search results for specific ESTs in the expression analysis**. Click here for file

Additional file 3 **Quantitative real-time PCR of transcripts differentially expressed in uninfected resistant and susceptible clones**. The putative functional genes from (a) to (h) were clearly discernible ESTs in susceptible libraries. The putative functional genes from (i) to (p) were clearly discernible ESTs in resistant trees. Elongation factor 1-alpha (EF1a) was used as the reference gene, and the data were calibrated relative to the transcript levels in resistant trees prior to nematode infection (at 0 dpi). The data are presented as the mean ± S.D. of three replicates. Means designed by the same letter did not significantly differ at *P *< 0.05 according to Tukey' s HSD test. Click here for file
