# Supplementary figures and images for: Characterization of resistance to pine wood nematode infection in Pinus thunbergii using suppression subtractive hybridization
Source: BMC Plant Biol. 2012 Jan 24;12:13. doi: 10.1186/1471-2229-12-13 (PMC3398268; doi:10.1186/1471-2229-12-13)

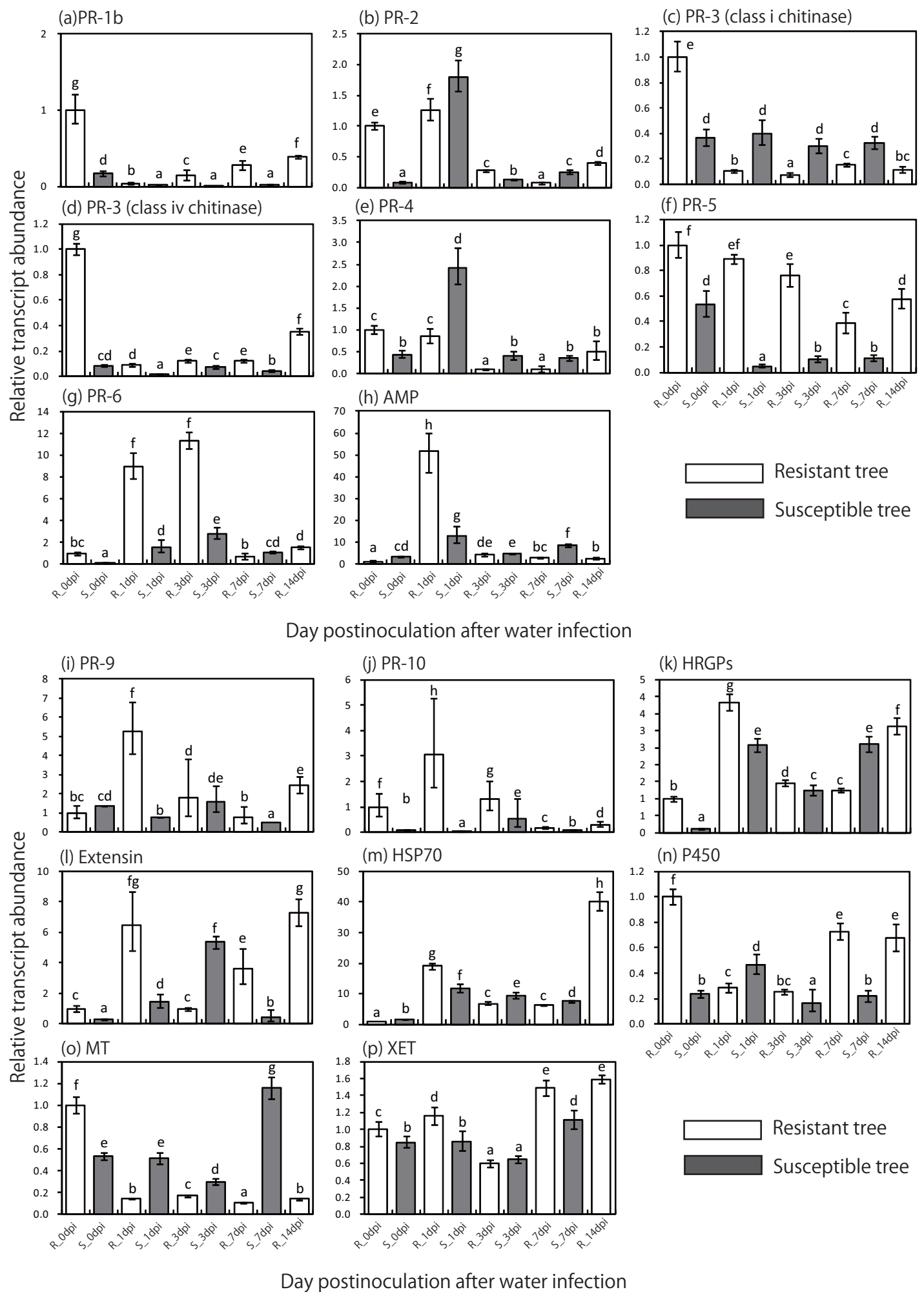

Supplement: Additional file 3 — Quantitative real-time PCR of transcripts differentially expressed in uninfected resistant and susceptible clones. The putative functional genes from (a) to (h) were clearly discernible ESTs in susceptible libraries. The putative functional genes from (i) to (p) were clearly discernible ESTs in resistant trees. Elongation factor 1-alpha (EF1a) was used as the reference gene, and the data were calibrated relative to the transcript levels in resistant trees prior to nematode infection (at 0 dpi). The data are presented as the mean ± S.D. of three replicates. Means designed by the same letter did not significantly differ at P < 0.05 according to Tukey' s HSD test. [file 1471-2229-12-13-S3.PDF]
